# Innovative Treatment for Cervical Precancer: A Qualitative Evaluation using the Theoretical Framework of Acceptability

**DOI:** 10.1007/s43477-026-00219-4

**Published:** 2026-03-24

**Authors:** Montserrat Soler, Gabriel Conzuelo Rodriguez, Javier M. Ayala, Karla Alfaro, Rene Novoa, Rachel Masch, Miriam Cremer

**Affiliations:** 1 Obstetrics and Gynecology Institute, Cleveland Clinic, Cleveland, United States; 2 Basic Health International, Pittsburgh, United States; 3 Basic Health International, San Salvador, El Salvador; 4 Basic Health International, Pittsburgh, United States

**Keywords:** Theoretical Framework of Acceptability, Cervical cancer, Cervical precancer treatment, Thermal ablation, Cryotherapy, Low- and middle-income countries (LMICs)

## Abstract

Acceptability is one of the most commonly assessed implementation outcomes, but there is little consensus on how the concept is defined or measured. The Theoretical Framework of Acceptability was developed to address this gap in the context of healthcare. This framework comprises seven constructs and has been applied across various settings, although work in low and middle-income countries remains scarce. Here we utilize the Theoretical Framework of Acceptability to evaluate the acceptability of cervical precancer treatment methods in El Salvador. Cervical cancer remains a leading cause of cancer death for women in low and middle-income countries. The World Health Organization has endorsed a triple strategy of vaccination, screening, and treatment to eliminate the disease as a public health problem. While the acceptability of vaccination and screening has received some attention, evidence related to treatment is limited. From November 2021 to May 2022, we conducted semi-structured interviews with patients participating in a clinical trial comparing three cervical precancer treatment protocols. Thematic and content analysis were used to extract salient themes from interview transcripts and themes were then mapped onto the framework’s constructs. All seven constructs were represented in the data, but two of these (affective attitude and intervention coherence) captured the most significant aspects of treatment acceptability in this population. In addition, some themes (i.e., pain, side-effects, stigma) were not adequately represented by existing constructs. We suggest an expansion of the Theoretical Framework of Acceptability to increase its generalizability.

Acceptability is one of the most commonly assessed implementation outcomes since it is expected to impact the adoption and sustainment of a given intervention (Proctor et al., 2011; Weiner et al., 2017). In the context of healthcare, however, there has been little consensus on the definition of the concept or its underlying dimensions (Bucyibaruta et al., 2022, 2023; Dyer et al., 2016). The Theoretical Framework of Acceptability (TFA) (Sekhon et al., 2017) was developed in response to this gap. The TFA is a multi-construct model for conceptualizing the acceptability of new interventions at various levels (individual, community, health system, etc.) and timeframes (prospectively, concurrently, or retrospectively) (see Fig. 1). The TFA has been used across various settings and conditions (see Paynter et al., 2023), although most studies in low and middle-income countries (LMICs) have focused on HIV-related interventions (Sekhon et al., 2021; Shayo et al., 2022; Young et al., 2024). The purpose of this study is to evaluate the acceptability of a novel cervical precancer treatment method in El Salvador.

**Fig. 1:**
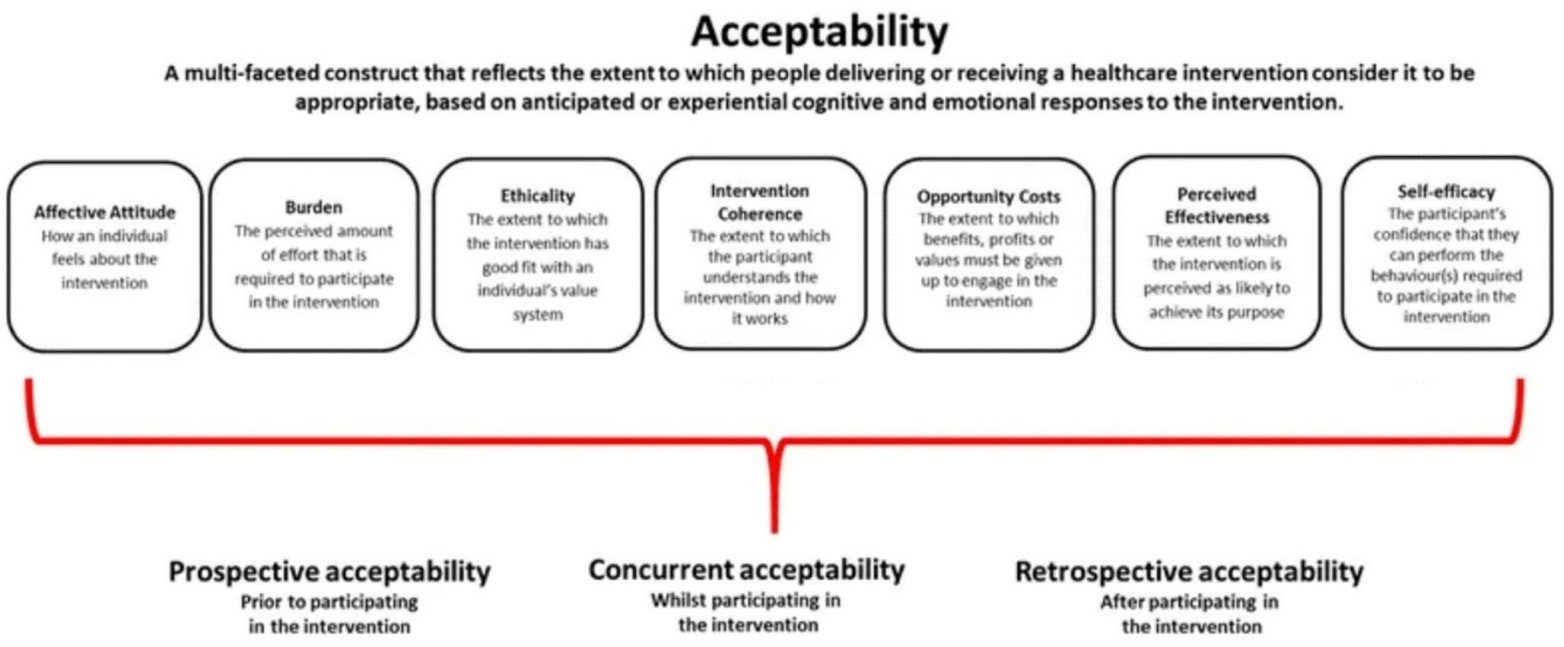
Depiction of the TFA with Corresponding Constructs (Sekhon et al., 2017)

Although preventable, cervical cancer annually kills 350,000 women around the world (Singh et al., 2023). Almost 90% of deaths occur in LMICs that do not have the infrastructure to support widespread prevention and early detection (Bray et al., 2024). To reduce this disparity, the World Health Organization has launched a call to action to eliminate cervical cancer by 2030 (World Health Organization, 2020). This initiative has highlighted the need for research on the implementation of human papillomavirus (HPV) vaccination, screening, and treatment interventions. While the TFA has been utilized to assess screening (Camara et al., 2022; Machado et al., 2024) and vaccination initiatives (Guillaume et al., 2023; Machado et al., 2024; Umutesi et al., 2023; Zhang et al., 2023), evidence on the acceptability of cervical precancer treatment methods has lagged behind with few exceptions (e.g., Camara et al., 2022).

Here we use the TFA to evaluate patient perceptions of two precancer treatments: gas-based cryotherapy, the most widely used method globally, and portable thermal ablation, a novel modality that utilizes a handheld device (Fig. 2). Both treatments apply extreme temperatures to the cervix using a probe to destroy precancerous lesions. Cryotherapy utilizes cryogenic gas to achieve freezing temperatures, but the need for gas presents well-known challenges related to cost, procurement, and transportation (Castle et al., 2017; Cremer et al., 2018; Maza et al., 2017). In contrast, portable thermal ablation devices are lightweight and operate on electricity or a rechargeable battery to generate temperatures of 100 °C. Cryotherapy also requires 11 min of treatment time, while thermal ablation is completed in 2–3 min (World Health Organization, 2013; World Health Organization 2019). The potential of thermal ablation as a portable, point-of-care alternative has rapidly moved the treatment from research to clinical practice (Boles et al., 2022; Petignat & Kenfack, 2020).

**Fig. 2:**
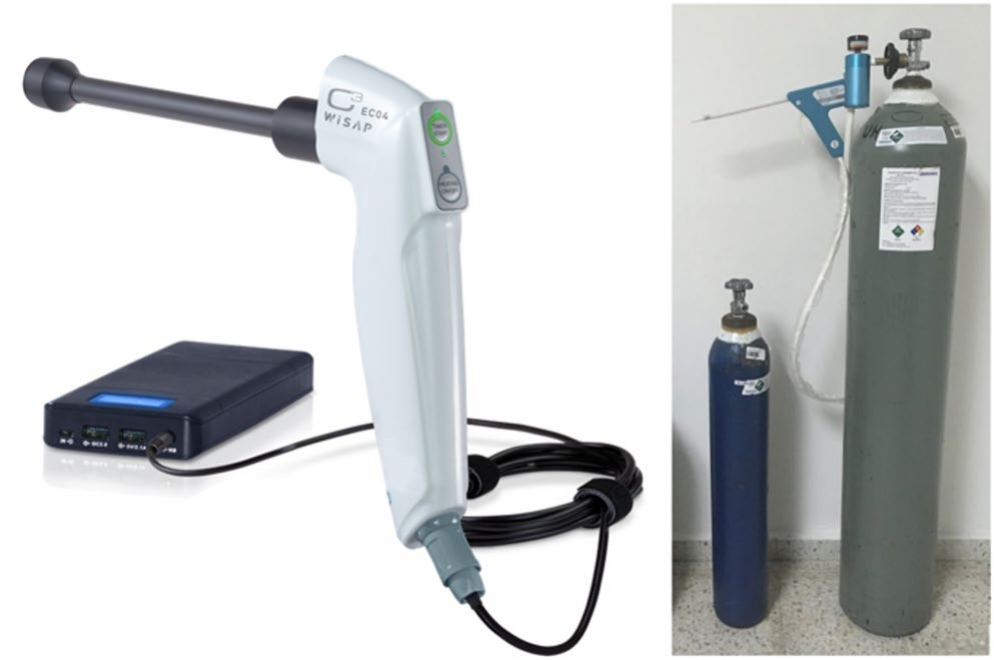
CIN2 + Treatment Devices. The handheld thermal ablation device with rechargeable battery weighs <2lb (left), while the cryotherapy device requires connection to a gas tank (right). A standard tank used for cryotherapy weighs 50-55lb and is approximately 35” tall

Thermal ablation was included in WHO guidelines for high-grade cervical precancer (cervical intraepithelial neoplasia grade 2 or higher or CIN2+) treatment in 2019, and this has resulted in the introduction of thermal ablation devices across various LMICs (Holme et al., 2017, 2020; Mwanahamuntu et al., 2022; Vallely et al., 2022). There is a growing literature on the feasibility of this treatment but, with few exceptions (Camara et al., 2023; Morse et al., 2022), most acceptability assessments focus on clinical safety and single-item questions regarding satisfaction with treatment or willingness to recommend the treatment to others (Metaxas et al., 2022; Sandoval et al., 2019; Sauvaget et al., 2022). Thus, this study will begin to fill gaps in the existing literature.

## Methods

The aim of this study was to evaluate the acceptability of thermal ablation treatment among women who had received it. In addition, we assessed the applicability of the TFA to a clinical intervention in a global setting. We followed a phenomenological design to explore participants lived experience of the treatment process. Data collection took place from November 2021 to May 2022 in the context of a randomized large-scale clinical trial (*n* = 1,152 women) comparing the performance of two thermal ablation protocols against gas-based cryotherapy using CO_2_ (NCT03429582). One protocol involved the use of a single ectocervical probe (one-probe protocol), while the other utilized an endocervical probe followed by an ectocervical one (two-probe protocol). Both devices were manufactured by WiSAP Medical Technologies GmbH (Brunnthal, Germany). To be eligible for the trial, women were over the age of 18, had no prior cervical surgeries or hysterectomy, and had a histologically confirmed diagnosis of cervical intraepithelial neoplasia grade 2 or higher (CIN2+). Thus, they had already undergone screening (HPV testing or cytology), colposcopy, and biopsy. Trial participation consisted of three visits: enrollment and treatment, 6-week follow-up, and final 12-month follow-up (enrollment is now completed and results are forthcoming).

The present research was designed as a nested sub-study taking place during both treatment and 6-week visits. This allowed us to assess acceptability immediately after treatment and some weeks later when women may be more aware of potential side-effects. Thus, target enrollment was a purposive sample of 15–20 women attending either the treatment visit or the 6-week follow-up of the trial and who were willing and able to participate in a semi-structured interview. We invited women from the three treatment arms to ensure representation of all methods. Interviews with healthcare workers were also carried out but only results from patient interviews are presented here. Interviews were conducted at a women’s health hospital in San Salvador where women were referred from various surrounding regions. At the time of data collection, cryotherapy was the only cervical precancer ablation treatment available in El Salvador’s public health system.

Interview guides consisted of 10 questions, each of which included several follow-up questions or probes. Questions were constructed as part of a broader project to investigate barriers and facilitators to cervical precancer treatment, including acceptability. Other themes included sociodemographic information and understanding of the cervical cancer screening process. Questions were not built using the TFA. However, during analyses it became clear that an acceptability framework would best help interpret results and the TFA was selected as a systematic and comprehensive model. Interviews were conducted individually at the end of each visit by native Spanish speakers, digitally recorded, and transcribed *verbatim* using specialized software (NVivo Transcription). Transcriptions were checked for accuracy and imported to NVivo Software v. 12 for coding and analysis using thematic and content analysis. Coding followed a combined deductive and inductive approach. An a priori coding scheme was developed based on previous data (Alfaro et al., 2015; Maza et al., 2018; Soler et al., 2022). Five randomly chosen transcripts were used to initially apply and refine the a priori coding scheme. Using an iterative process, the lead author coded the remaining transcripts, refined the coding scheme as needed, and generated overarching themes. Another Spanish-speaking team member then utilized the final coding scheme to independently code a random sample of four transcripts. NVivo was used to calculate agreement percentage in the sub-sample of transcripts, which ranged from 73.1% to 100% with an average of 94.37%. Both team members reviewed discordant codes and themes until consensus was reached. Themes were mapped onto TFA constructs to evaluate dimensions of acceptability of the treatment interventions. Selected quotes were chosen to illustrate major themes (quoted women with the same age and treatment are differentiated by a superscript letter next to their age).

### Informed consent

was obtained from all participants. All procedures were approved by the Cleveland Clinic Internal Review Board (20–1128) and by the El Salvador National Ethics Committee for Health Research CNEIS (2020/08). Data is available upon reasonable request.

## Results

Thematic saturation was reached at 18 participants. Of these, 4 women had undergone cryotherapy, 6 thermal ablation with a one-probe protocol, and 8 thermal ablation with a two-probe protocol. Half of the women (*n* = 9) were interviewed the same day their treatment took place and the other half (*n* = 9) at the six-week follow-up visit. Demographic and background characteristics of the women are summarized in Table 1.

**Table 1:** Sociodemographic Characteristics of Participants

Variable	
Age, mean (SD) Range (min., max.)	36.83 (4.36) 31, 48
Years of formal education, mean (SD) Range (min., max.)	7.33 (5.26) 0, 18
Household size, mean (SD) Range (min., max.)	4.38 (1.53) 2,7
Occupation, n (%)	Homemaker = 9 (50%) Domestic worker = 2 (11%) Informal commerce = 4 (22%) Coffee farmer = 1 (6%) Line cook = 1 (6%) Administrative assistant = 1 (6%)
Marital status, n (%)	Married/partnered = 12 (67%) Single, divorced or widowed = 5 (27%) Missing = 1 (6%)
Residence area, n (%)	Urban = 44% (8) Rural = 56% (10)
Travel time to hospital, in min., mean (SD) Range (min., max.)	52.22 (31.27) 10, 105

TFA consists of seven constructs shown in Table 2. All were represented in the data. The most frequently coded themes corresponded to the “affective attitude” and “intervention coherence” constructs. These were referenced multiple times by all participants and reflected a time-dependent dimension (before and after the treatment intervention). Constructs and corresponding themes are described in detail below (starting with the most salient).

**Table 2:** TFA Constructs and Corresponding Interview Themes

TFA Constructs	Themes
Affective attitude *How an individual feels about intervention.*	Fears regarding diagnosis and treatment (including fear of cancer, expectation of pain, etc.) Uncertainty (not knowing what diagnosis meant, what treatment implied, etc.) Relief after treatment
Intervention coherence *Extent to which participant understand the intervention and how it works.*	Understanding of HPV and cervical cancer Understanding of treatment
Ethicality *Extent to which intervention is a good fit with individual’s value system.*	Satisfaction with overall experience Attending treatment is good for self/others (for reasons other than efficacy) Quality of care Discomfort with male providers
Perceived effectiveness *Extent to which intervention is perceived as likely to achieve its purpose.*	Belief that the treatment will cure the lesion
Self-efficacy *Individuals’ confidence that they can perform behaviors required to participate*	Confidence in following post-treatment recommendations
Opportunity costs *Extent to which benefits*,* profits*,* or values must be given up to participate.*	Childcare (during and after treatment) Work and domestic activities (during and after treatment)
Burden *Perceived amount of effort required to participate.*	Travel/transportation Convenience (treatment duration, ease, outpatient procedure)

At the time of the interviews all patients had already undergone treatment. Post-treatment recommendations and repeat screening were discussed during the treatment visit as part of patient counseling

## Affective Attitude

Prior to the intervention, participants’ feelings reflected anticipated fear and anxiety related to treatment (regardless of type), diagnosis, or a mixture of both (34 references across all patients). Regarding treatment, salient themes were uncertainty as to the nature of the procedure and the expectation of pain:

Ah, very bad from nerves, cold. I got a headache, when I went to the bathroom I felt sick, more sick, what can I tell you, for a moment I did not want them to do that to me, because I said, oh my God, who knows what they’re actually going to do to me? I was very worried, I even felt like throwing up. (34y, termal ablation two-probe protocol)

Never, I had never had that done, I don’t know, how will it go, how will it feel or maybe it will hurt and I will not be able to stand it. (41y, thermal ablation one-probe protocol)

In terms of diagnosis or prognosis, responses often reflected uncertainty of what their HPV test or biopsy results meant or fear of having cancer:

They [fears] were, what they were going to tell me, what it was that I had, what did I have, and all of that, really. That was my nervousness. (34y^a^, cryotherapy)

Well, they told me that it was positive, and I said, I already have cancer! (39y, thermal ablation two-probe protocol)

On the other hand, women’s affective attitude toward the intervention once the treatment visit had concluded or during the 6-week follow-up reflected a significant change. Most described feeling much calmer and expressed satisfaction at having received treatment:

Now today I feel a lot more calm, with the explanation I received and all of that, with the treatment I mean I feel that I leave more calm, I mean, it looks like negative, I expected to be crying in the hallways, but thank God, no. And the nerves, I mean, compared to how I came in, I feel like I’m better. (33y, thermal ablation two-probe protocol)

Bad, super bad. Before coming here? Yes, of course, because when they tell you that something bad has come up, the only thing that one can imagine is that I will die of cancer, or something like this, but it really is about educating oneself. Because part of what I understand now is that there are different types of papilloma and there are some that, I mean, they are bad, but some are treatable. (35y, thermal ablation two-probe protocol)

## Intervention Coherence

Intervention coherence refers to what or how much participants understood about the intervention. Two main themes aligned with this domain: awareness or knowledge of HPV and cervical cancer (discussed by 16 women, 22 references) and understanding of the treatment itself (discussed by 18 women, 29 references). Despite all women having already undergone biopsy as an eligibility requirement for the trial, most expressed uncertainty and misunderstandings regarding both their diagnosis and the treatment they received. Only 3 women mentioned having awareness of cervical cancer prior to their positive screening result: one because she regularly attended a women’s clinic, another because she had undergone screening in the past (i.e., cytology), and another because she had a relative with cervical cancer. In contrast, 8 women had never heard about cervical cancer before their diagnosis:

Well, I have only heard of cancer, but I don’t know how this would be, I wouldn’t know what to tell you. (48y, thermal ablation one-probe protocol)

As described above, a few women expressed that they had learned more about HPV and cervical cancer after their treatment visit. However, most women were still unsure as to the nature of the disease (i.e., where it came from, what part of the body was treated, difference from other cancers, etc.). Questions about current knowledge of HPV and cervical cancer elicited answers that included, “I have no idea” and “I don’t remember”, “I wouldn’t know how to explain it”, and “Well, among the things I have heard is that it comes about from so many infections, more or less, but I didn’t understand a lot, no”. In terms of treatment, only 2 women had previously heard of cryotherapy (“congelo”) but were uncertain of what it entailed. Most had trouble recalling the name or type of treatment they received and at least one was concerned that the treatment would harm her uterus:

Yes, yes, well, it is always a bit difficult to understand the medical names of things, right, for example, here, I’m already confused, the procedure they just did, if it was as they say cold or hot, I’m already confused. (33y, rural, thermal ablation two-probe protocol)

I forgot. The first one I don’t remember with what it was [with cold or with heat] no…but they told me that it was because I had little stain in the neck of the uterus. (39y, thermal ablation two-probe protocol)

Yes, a, a burn…yes, they told us that was so that something did not expand, what I had. (32y, thermal ablation two-probe protocol)

With respect to the treatment, well there you were, seeing that they were explaining everything to us step by step what it really was. Honestly, I did not understand very well, but I understand that I am in professional hands, and well, I need the treatment. (35y, thermal ablation two-probe protocol)

To prevent, but I hope it is not that they will take something from my uterus…because like here, there are some that they take a small piece of their uterus, they take, what is it called, the neck. And then, because in the neck is where the cancer is, but even then, then it is not the same. (34y^a^, cryotherapy)

Five women who expressed a clearer understanding of treatment had sought information online before their appointment, either by themselves or with the help of a close relative. These women also reported higher-than average formal education (1 had post-high school education, 3 had at least some high school education, and 1 had completed middle school):

Well, she [daughter] started to investigate a bit about the topic and told me, explained to me that there were two types [of treatment] that they could give me, right. And well, she told me it was a treatment to avoid a cancer. (42y^a^, thermal ablation one-probe protocol)

## Ethicality

The TFA defines ethicality as the extent to which an intervention is a good fit with the individual’s value system. The themes that most closely aligned with this construct were satisfaction with the overall experience and a perception of the treatment as “good” for reasons that did not explicitly referenced efficacy (e.g., it is good to take care of oneself, it is good to know what one has, etc.):

Well, yes, that’s my thinking, really, since the doctor started doing that, well I said, it’s very excellent what they’re doing to me. And I will invite my friends to, to come and do that, because it is very important, because the reality is that before I didn’t take care of myself, because I even said, I’m only trash, not worth anything, I mean, all my life I’ve had that, but thank God, ultimately, well, I am worth a lot and health comes first. (34y, thermal ablation two-probe protocol)

Well, one part happy, because imagine that, I saw that I had it for a while and also I thank God that they called me for this treatment that they were going to give me, that way one knows what one has. (40y, thermal ablation two-probe protocol)

Before the disease or letting things grow, the best thing is to undergo tests, not, not stay behind. Not ignore. And not only because this day I have an appointment for this, or a treatment, but to get to know a little bit more of the disease before it gets to us. (31y, thermal ablation two-probe protocol)

Another salient theme that reflected patients’ values were perceptions of how they were treated by providers and staff. Eight women referenced trust or gratitude towards the healthcare providers involved in their care and mentioned positive interactions with staff as an important aspect of their experience:

Um, it shouldn’t be, but basically the friendliness and respect that we are treated with, it doesn’t matter how one is dressed or who you are. That more than anything. They gave us transportation from [redacted] to here…and here, they have explained everything step by step. And I feel it is really nice because they have treated us, well, decorously, like any human being should be treated. (age 35y, thermal ablation two-probe protocol)

That’s what I told my work colleagues that the treatment from the first appointment that I had, everyone was super attentive. The place, where they did the colposcopy, very clean, the nurses’ manner, sometimes one finds nurses that are rude, right, so I felt that the experience was very gratifying. (34y^b^, cryotherapy)

On the other hand, a part of the experience that challenged a woman’s values was the need to undergo a gynecologic exam by a male provider:

I was embarrassed because I was going to be touched by a male, then I felt like almost, because a doctor had never seen me, I have had exams, but not like that, how I was. So then that was my embarrassment, that the doctor saw me, but ultimately it felt normal, when he was examining me I no longer felt like I was nervous. (34y^a^, cryotherapy)

The discomfort of the speculum exam, particularly when carried out by a male provider, is a theme that has emerged in previous studies as a potential barrier to screening (Laskow et al., 2017; Maza et al., 2018) and which has also been found in other settings (Lau et al., 2022).

## Perceived Effectiveness

In a similar population, some women have expressed doubts regarding ablation treatment efficacy and reported that they would only feel more confident after their follow-up appointment (Soler et al., 2022). However, women in this group were generally optimistic that the treatment would work, as described below:

After the treatment and everything, I have felt, well, I’ve felt more calm, I’ve felt more calm because I see that, thank God, that with this treatment already it has worked for me, and it’s good. (34y^c^, cryotherapy)

Yes, they explained it, because they told me that I had a lesion and that with that [the treatment] it would be enough. Yes. So really, my advice is for anyone who feels that way, that does the check-ups and ends up with a lesion like that, my advice is to continue, continue with the treatment, because it is excellent. (42y^b^, thermal ablation one-probe protocol)

Since I came and started to see, it has been a pretty new experience, and I saw that the treatment, we work towards something good, toward something positive, to see if, well, hoping that in the next appointment, it will be something that won’t be there anymore, that it won’t continue, that with this we can say that the disease won’t be there. (41y, thermal ablation one-probe protocol)

### Self-Efficacy

Self-efficacy is defined as individuals’ confidence that they can perform behaviors required to participate in the intervention. All interviews were conducted after women had already completed the treatment itself, but an important part of the intervention is following post-treatment recommendations. These include sexual abstinence and avoidance of douches, tampons, immersion in water, and physical exertion for four weeks. At the treatment visit, all women expressed high confidence in their ability to adhere to recommendations. However, interviews at the follow-up visit revealed difficulties with sexual abstinence, a barrier reported in other settings (Bradley et al., 2006; Coffey et al., 2005; Wilson et al., 2021), and with avoiding some types of physical effort:

The sexual one only [was difficult], but I feel that that one is more common among all the women who have treatment. But if people love us, they have to be part of our support. (35y, thermal ablation two-probe protocol)

Well, me, my husband is more, how can I say it, he’s careful because he understands, we talked about it, we talk and he listens and pays attention. He isn’t, how can I say it, because some are very unruly, but well, he’s not. (34y^a^, cryotherapy)

Well, the one about doing, the physical effort to put it that way [is difficult]. Because the one about the sexual relations that they tell us to not do, one knows that no, and I tell my husband, right, because even if it sounds bad, but there are other ways because remember that as a woman one is different from a man, a man has a lot of needs. With us it can be years maybe and stay the same. So then I’ve tried to help him in other ways, without having to [have sexual relations]. (37y, thermal ablation one-probe protocol)

The one [recommendation] I maybe did not follow a lot was probably the one about work. Yes, because I had to. But in 15 days they said that whatever I could do, that I should see. After that I started working but in a way that I didn’t do a lot of physical force, right? We would cut [coffee plants] but my son would pull the coffee out. (41, termal ablation one-probe protocol)

## Opportunity Costs

Two themes emerged as opportunity costs of the intervention at different stages. With regard to the treatment visit, three mentioned the need for childcare as a potential barrier to attend. In addition, following post-treatment recommendations, as mentioned above, and the side-effects of treatment impacted some women’s ability to complete their daily activities:

When I had another procedure [biopsy], that they gave me the same 40 days and I couldn’t make any effort or anything like that, I talked to my sister-in-law and she helped me, because where we live, that is daily life, pull water, take water from the well. In fact, a lot of people go and look for firewood and they carry their big firewood bundle, right? I don’t get firewood because we use gas, but sometimes I do have to take water from the well, carry it, or if they come to deliver water, the same, one has to carry it. So you have to make physical effort. (37y, thermal ablation one-probe protocol)

My work colleagues, they told me that, well, one in fact said ‘after I had the treatment I couldn’t walk, I was lying down, and you came to work like nothing. So I think that affected you’, she told me, because actually there were two days when I felt a lot of pain, they sent me home from work, they said, you’re not really doing anything, better go home. (34y^b^, cryotherapy)

## Burden

The TFA describes burden as “effort” to participate in terms of time, expense, or cognitive or physical energy required. Fifteen references across 10 women mentioned transportation to the treatment center as a difficulty, due to cost, long travel times, or requiring navigation in an unfamiliar city. However, most women received transportation as part of trial participation and there were no direct costs of treatment, so these themes were likely underrepresented. In terms of time, two women assigned to cryotherapy referred to the treatment as uncomfortable long (cryotherapy lasts approximately 11 min compared to 2–3 min for TA). On the other hand, 6 women that received thermal ablation mentioned the short treatment time as an advantage:

Wow I said, how quick!…I never imagined it would be like this, I thought it would be worse for me. (34y, thermal ablation two-probe protocol)

I was nervous but the process was light…well, my nervousness was the reason, what I had, really, and I thought that the treatment was longer, but not, seeing it properly, it is short. (37y, thermal ablation two-probe protocol)

I mean, it’s not even to come and stay in the hospital, rather it’s only a couple of minutes. (33y, thermal ablation two-probe protocol)

### Other Themes

At least two themes emerged in interviews that did not fit into the TFA’s existing domains, although they overlapped with some of them (i.e., burden, ethicality, self-efficacy): physical harms and stigma. Harms were represented by descriptions of pain during the treatment (in contrast to anticipated pain) and side-effects in the following weeks. Pain during treatment was described by all women as tolerable:

The pain is tolerable, as they say, I thought it was going to hurt more. But no. (32y, thermal ablation one-probe protocol)

From what I knew, I expected [the pain] to be stronger, much stronger, but no. It was only in the moment and that was it, because then later the pain settled and that was it. (31y, thermal ablation two-probe protocol)

I felt a little [pain], slowly, but it wasn’t, it wasn’t much. (48y, thermal ablation one-probe protocol)

In terms of side-effects, all nine women interviewed at the follow-up visit experienced abdominal pain or cramping for several days after the procedure and vaginal discharge for up to a month. Mild bleeding or spotting was mentioned in three cases, but pain and discharge were the most intense and uncomfortable and sometimes interfered with daily activities (thus resulting in opportunity costs as mentioned above):

The next day when the sun came up, right, I felt as if I had had a child, something there, my womb hurt. But it was from the same thing, my mom said, but I couldn’t go to work. But when, any little thing I did, when I got up or something like that, it was a lot, a lot of liquid came out, yellowish it was. (41y, thermal ablation one-probe protocol)

I was about to continue with my [high school] studies because they have them Saturday and Sunday, right, but like, because of the recommendations, I didn’t want to talk too much, because it felt like it hurt to walk. (34y^c^, cryotherapy)

That same day in the afternoon it was normal and the next day was normal, and two or three days later was when I started feeling pain, a pain in my womb as if I was going to be my period…like a cramp, it was two days that it was pretty intense. (34y^b^, cryotherapy)

[The discharge] lasted almost like 25 days…it felt bad, yes…I told my daughter to call and ask. And she asked and she said you told her it was normal and that about a month it would last. So I was resigned, I said, it will go away. (39y, thermal ablation two-probe protocol)

The other theme not captured by the TFA was stigma. The two most frequent manifestations were shame associated with a positive HPV status and fear of disclosure to others, especially husbands or partners:

I wanted to handle this like more privately, to put it that way, because for reasons that sometimes in the cantones [rural communities] people can be badly intentioned… for my part, I don’t feel proud, really, to be with this little problem right now. (33y, thermal ablation two-probe protocol)

In the first moment I was scared, and also I worried, because how do I tell my husband all this? Because he was not responsible, and what does that mean, that means I gave it to him, I mean, it’s very contradictory. So the doctor told me to explain it with other words, being the same thing. So that’s what I did. I mean, my husband doesn’t know for certain directly that it’s the virus that I have, I told him in sort of another way…I can’t tell him, look, the other one gave it to me and now I gave it to you and I didn’t even know. (37y, thermal ablation one-probe protocol)

Until now I haven’t told my partner. I’ve just told him that I’m doing a treatment but I haven’t told him what it is about because I’m ashamed, that he will judge me or say something, that he will be upset, I don’t know, because ultimately he’s my new partner. (35, thermal ablation two-probe protocol)

## Discussion

Acceptability is a crucial outcome of implementation research (Proctor et al., 2010) particularly at the early stages of developing or evaluating an intervention (Damschroder et al., 2022; Reilly et al., 2020). It is also one of the most frequently assessed concepts in effectiveness and implementation studies: a quick PubMed search reveals 31,816 articles with the keyword “acceptability” in 2025 alone (in contrast, there are 4,185 mentions of “fidelity” in the same period). Despite its widespread use, there is no consensus on how acceptability is defined, and it is often used interchangeably with other concepts such as satisfaction, appropriateness, or convenience (see Paynter et al., 2023; Sekhon et al., 2017). Thus, the TFA is an important theoretical tool that explores the multi-faceted nature of acceptability and provides a systematic model to operationalize and measure it.

In this study, the TFA was able to capture most, but not all, aspects of acceptability of a cervical precancer treatment. The most salient of the posited dimensions of acceptability were affective attitude and intervention coherence. These were interrelated and time-dependent: prospectively, poor intervention coherence led to negative affective attitudes. Although women in this study had already undergone screening and cervical biopsy, the majority experienced significant distress prior to their visit due to uncertainties regarding the meaning of their diagnosis and details of the treatment process. Retrospectively, however, women viewed the treatment much more positively. The TFA’s inclusion of a temporal component was relevant for understanding how these constructs changed over time, although health literacy surrounding HPV and cervical cancer remained low. Multiple reviews have found that lack of awareness and education are significant barriers to screening and precancer treatment globally (Gillaume et al., 2020; Mantula et al., 2024; Petersen et al., 2022). Thus, there is a clear need for initiatives that inform, reassure, and ultimately facilitate women’s attendance to preventive visits. Other dimensions that captured barriers to treatment included opportunity costs (e.g., childcare, work) and to a lesser extent, lack of self-efficacy (i.e., difficulties in following recommendations). Facilitators, on the other hand, were perceived efficacy of the treatment and ethicality (i.e., treatment being good for the self or others, quality of care by providers).

The study sheds light on the acceptability of thermal ablation compared to cryotherapy as the former rapidly gains ground as a cervical precancer treatment. There were no significant differences in the frequency or saliency of themes across any treatment modalities, with the exception of some participants commenting positively on the short duration of thermal ablation (corresponding to the burden construct). Nevertheless, most women perceived both treatments as low burden overall (i.e., outpatient, short duration). While evidence indicates that thermal ablation is more painful than cryotherapy (Soler et al., 2022) and there are reports of thermal ablation causing severe pain in a minority of patients (Basu et al., 2024; Metaxas et al., Mungo et al., 2020), the women in this group did not mention pain as a significant concern. There were also no differences in pain or discomfort among women treated with a single thermal ablation probe and those who underwent the two-probe protocol, which could ostensibly be more painful due to the placement of an additional narrow probe in the cervical opening.

Side-effects, on the other hand, were mentioned often and could be sufficiently disruptive to impact everyday activities. This has been previously noted as a potential barrier to treatment acceptability that deserves more attention (Gottschlich et al., 2023). Neither pain nor side-effects, broadly categorized as physical harms, were captured by the TFA. Other authors have argued there is a need to expand the framework to include clinical adverse events to maximize its utility in healthcare implementation studies (Payne et al., 2023). Stigma was another theme that did not fit into the TFA’s existing domains. Stigma has been reported as an important and understudied barrier to cervical cancer screening (Morse et al., 2023; Paneru et al., 2023; Peterson et al., 2021). A key feature of stigma is that it is defined by the social group, but an individual can experience stigma without sharing the values of those who enact it. While the TFA ethicality construct considers an individuals’ own values, external social forces (i.e., family influence, peer pressure, coercion) may also be powerful drivers of the acceptability of an intervention, particularly in populations where strong communal ties are a central feature of everyday life. Given these findings, we suggest additional constructs to expand the applicability of the TFA (see Table 3). The uptake of thermal ablation or other innovations in healthcare largely depends on patient’s preferences and perspectives. Thus, it is essential to develop tools that can operationalize and measure concepts such as acceptability so that evidence-based interventions can be successfully incorporated into global health systems.

**Table 3:** Proposed Expansion of TFA Constructs

TFA Constructs	Definition
Affective attitude	*How an individual feels about intervention.*
Intervention coherence	*Extent to which participant understand the intervention and how it works.*
Ethicality	*Extent to which intervention is a good fit with individual’s value system.*
Perceived effectiveness	*Extent to which intervention is perceived as likely to achieve its purpose.*
Self-efficacy	*Individuals’ confidence that they can perform behaviors required to participate.*
Opportunity costs	*Extent to which benefits*,* profits*,* or values must be given up to participate.*
Burden	*Perceived amount of effort required to participate.*
Harms	*Adverse effects of an intervention experienced by participants (side-effects*,* injuries*,* etc.)*
Social pressure	*Social forces that influence a participant’s willingness to participate in an intervention (i.e.*,* peer pressure*,* stigma*,* coercion*,* etc.)*

Adverse events may be physical or psychological

### Limitations

This qualitative assessment has several limitations. The sample was small and not representative of the patient population. Women who participated in interviews were recruited as part of a clinical trial and some received transportation to the clinic, which may have favorably impacted their perceptions of the treatment compared to those who receive it in routine clinical practice. In addition, study providers had extensive research experience and training and treatment procedures were carried out in a large hospital specialized in women’s healthcare. In the clinics where treatment is normally carried out, providers face competing priorities, heavy caseloads, and more limited resources. The constraints of the public health system will impact patient experience in ways that cannot be captured in this study. It is also important to mention that the barriers and facilitators of treatment acceptability uncovered in this study are specific to our participants. There may be many other factors that influence both patient and provider preferences, particularly in different sociocultural contexts, but those were not found in this sample.

## Conclusion

In implementation science, acceptability of new interventions is a central but poorly defined and measured concept. The TFA is a useful theoretical model because it defines acceptability as comprising seven distinct constructs. This allows for clearer and more systematic assessments of a critical concept that impacts adoption and maintenance of new interventions. Here, the TFA was used to evaluate acceptability of cervical precancer treatments in El Salvador. Findings revealed that the TFA was able to capture most barriers and facilitators of acceptability expressed by women who received treatment. The temporal dimensions of the TFA and two constructs, affective attitude and intervention coherence, were salient in this population. However, there were themes related to physical harms and social pressures that were not captured by the TFA. An expansion of the framework to include new constructs may be necessary to enhance its generalizability in healthcare settings.

## Data Availability

Data is available upon reasonable request.
